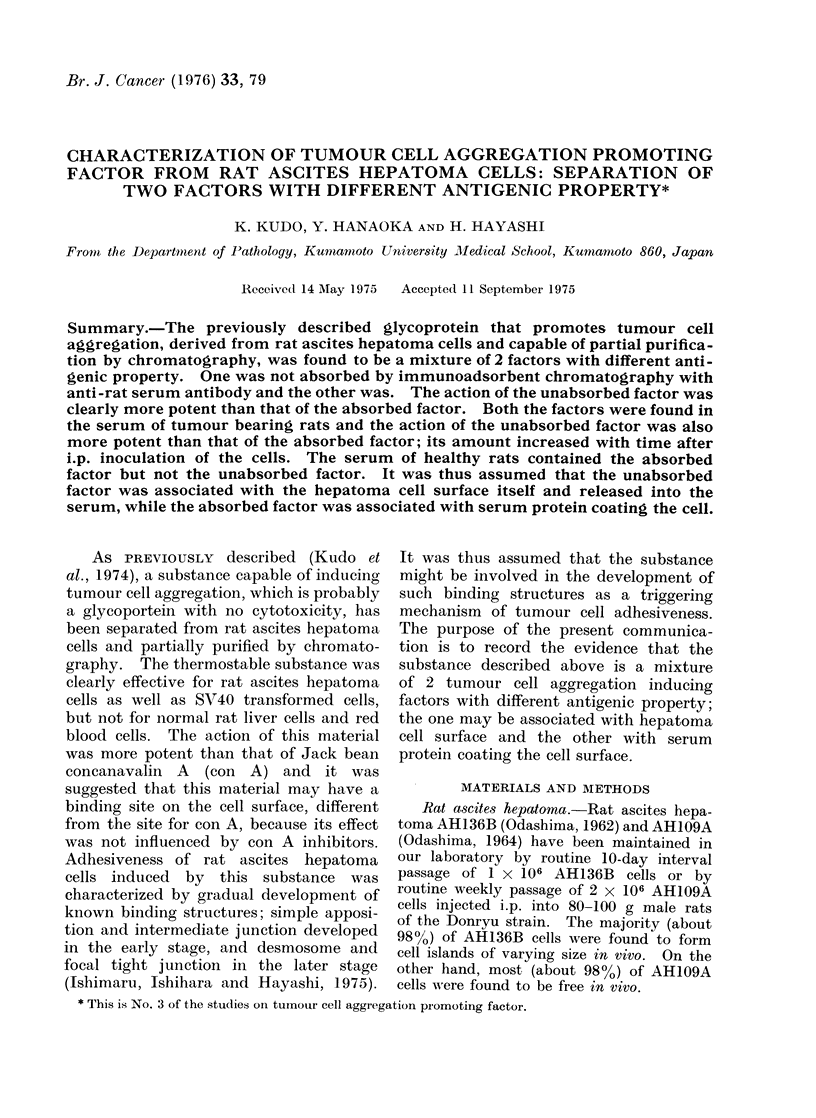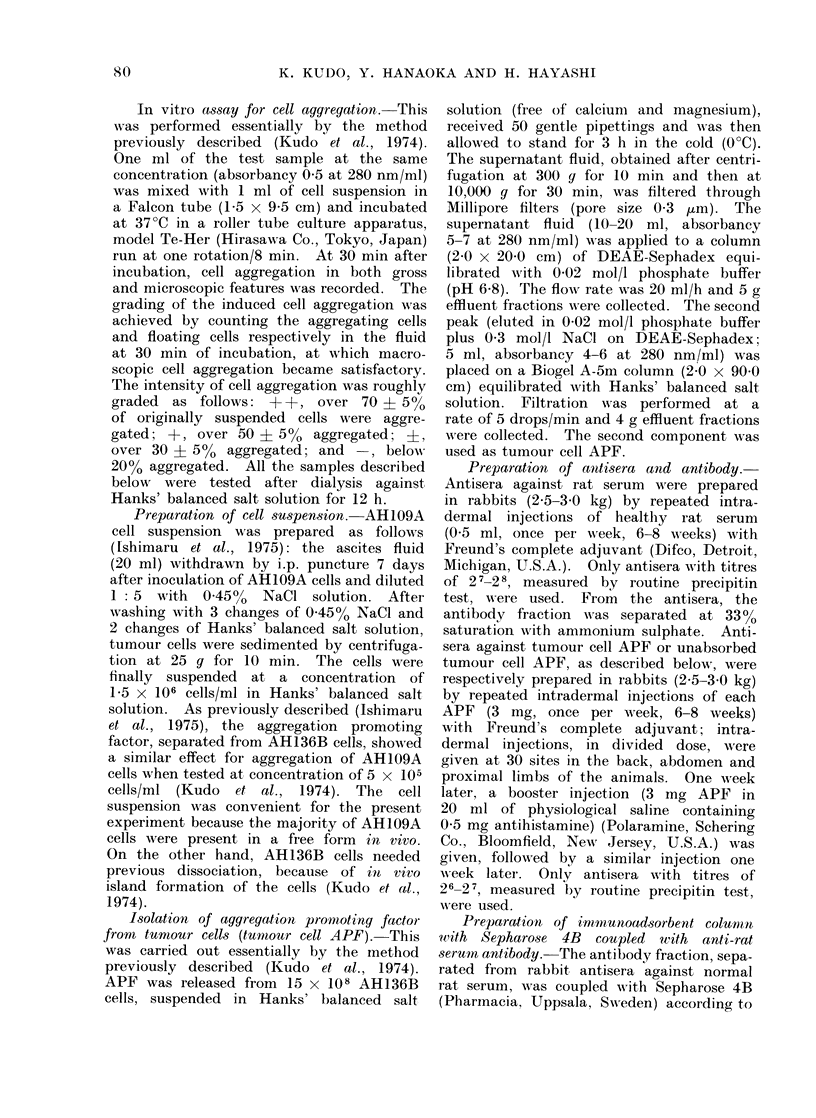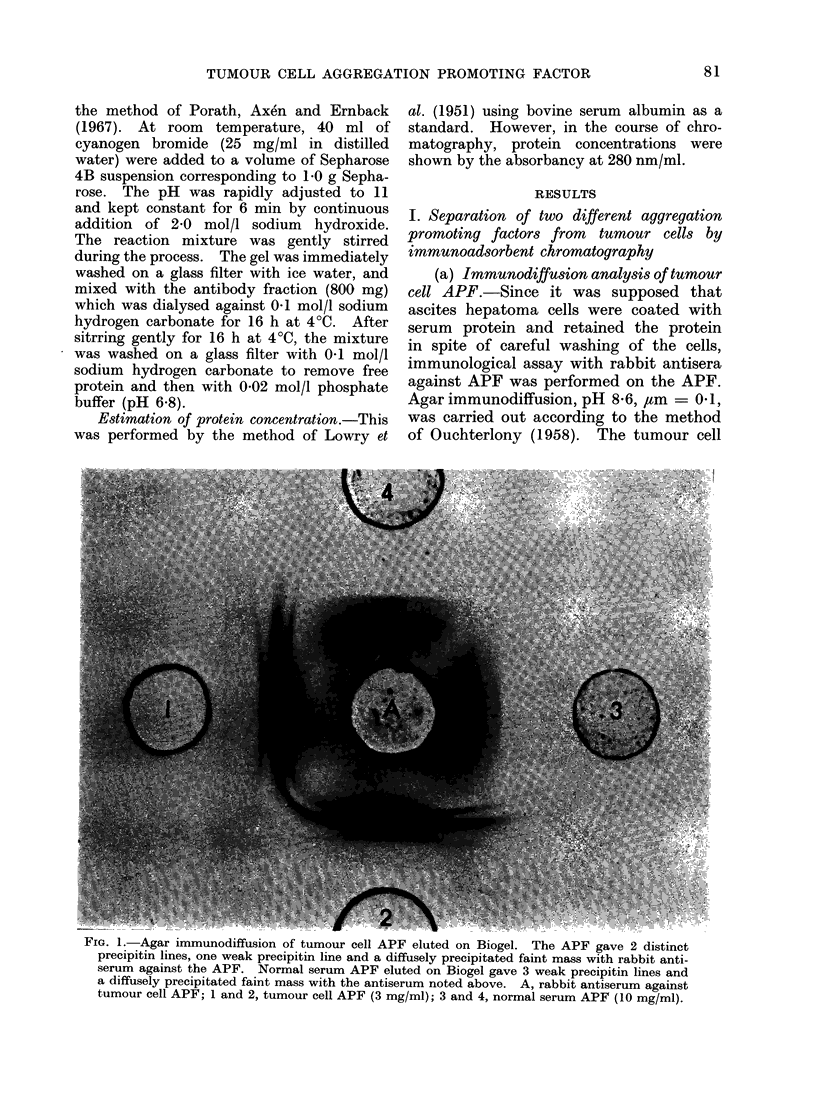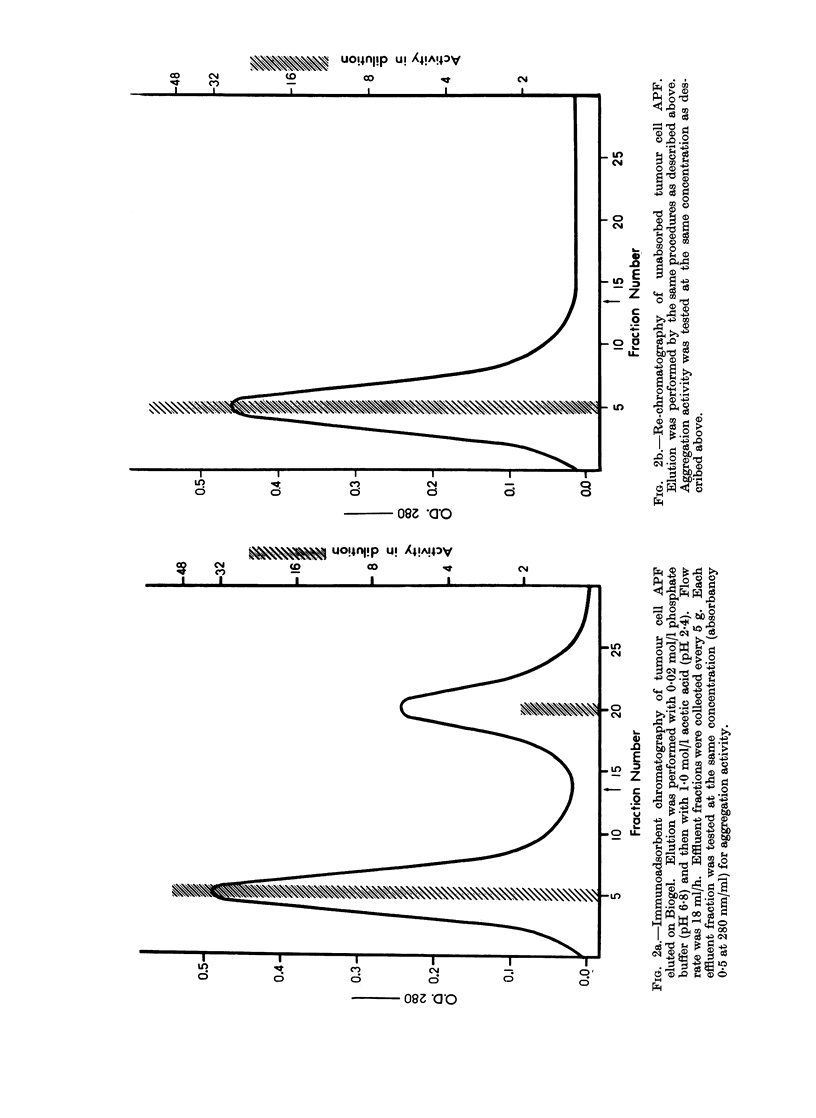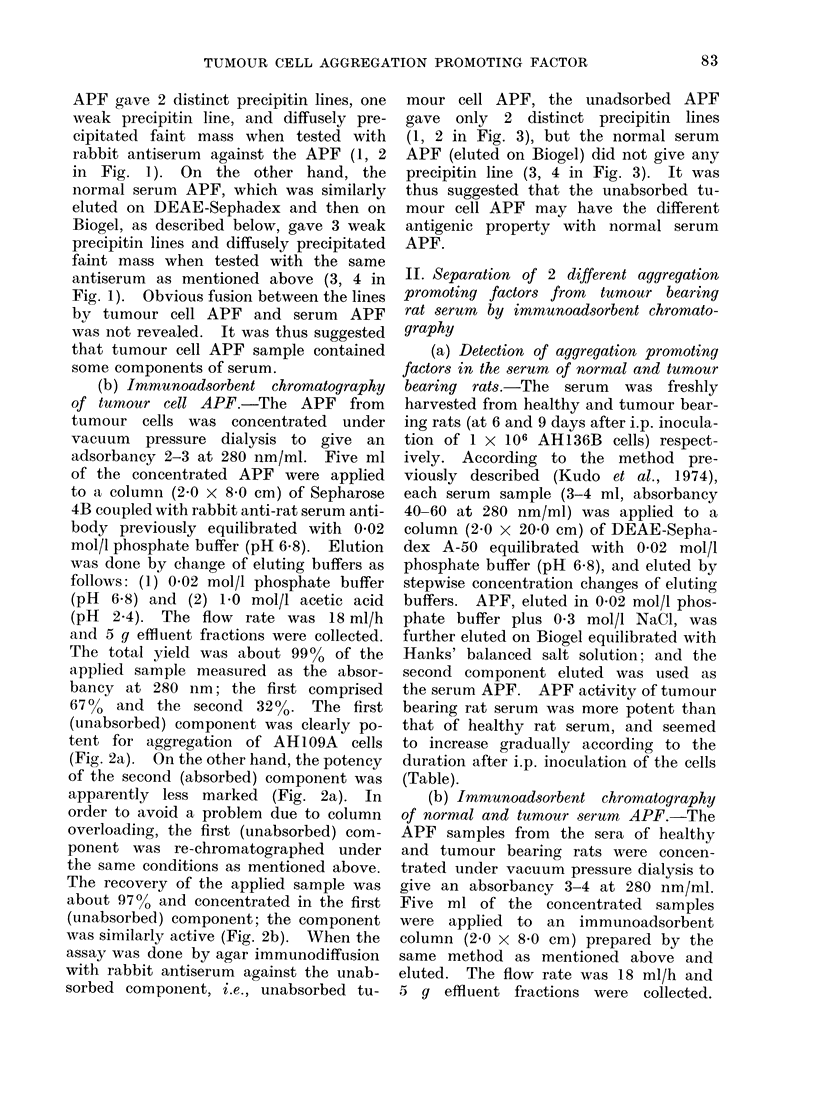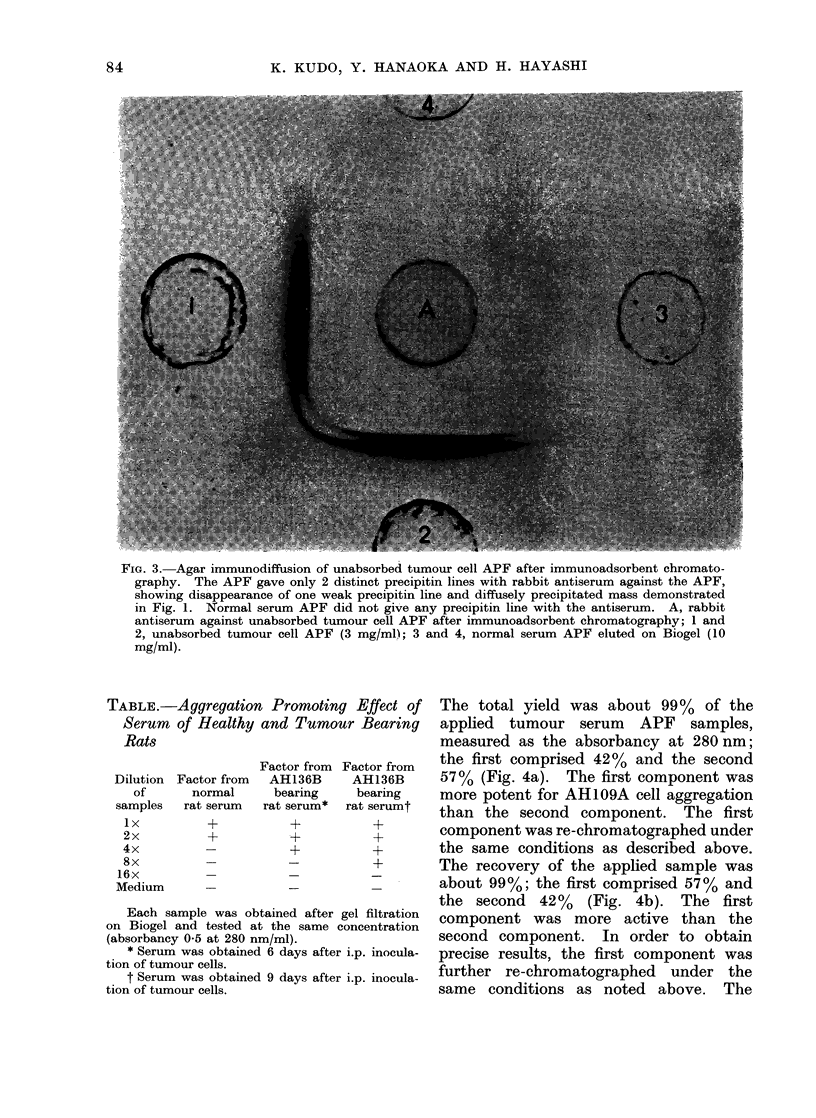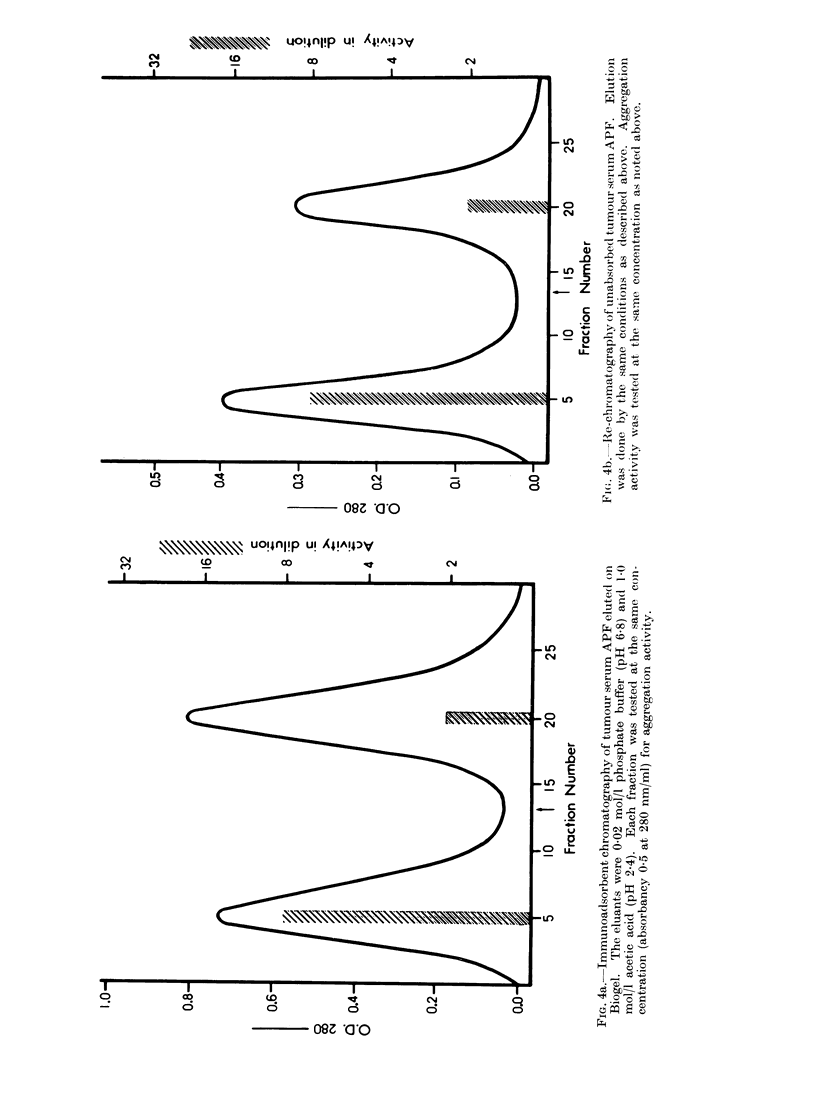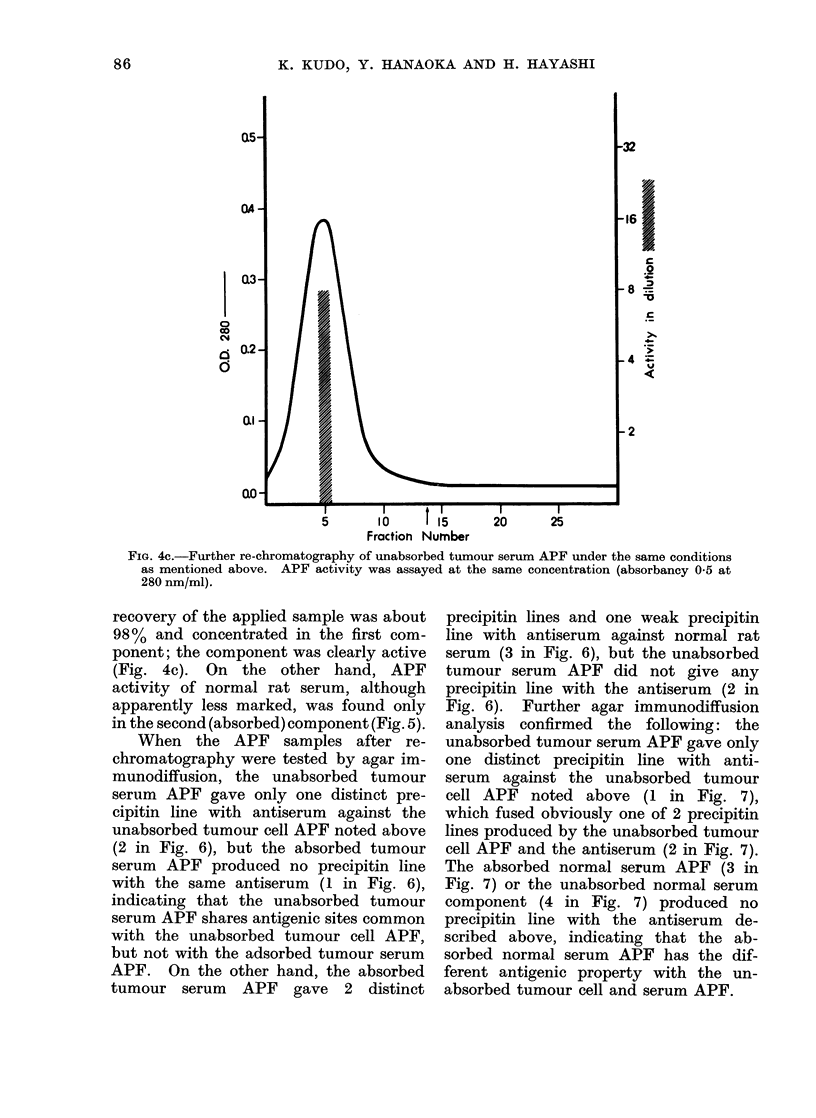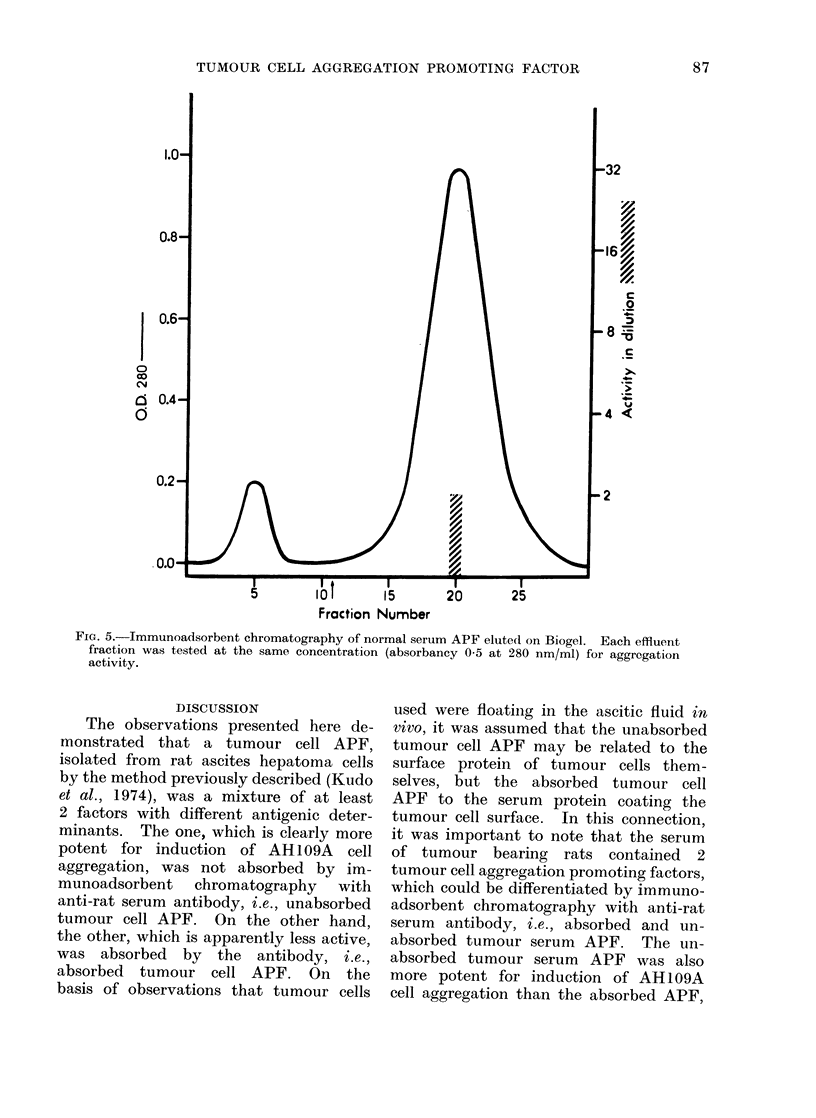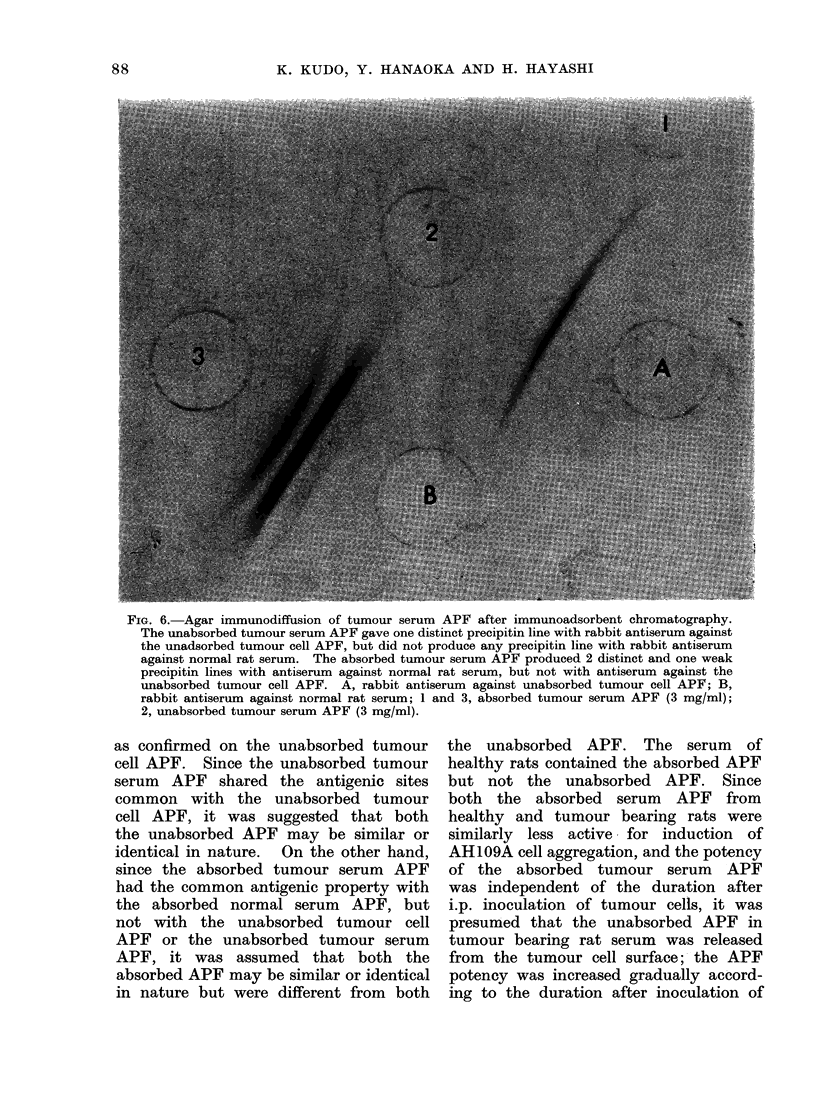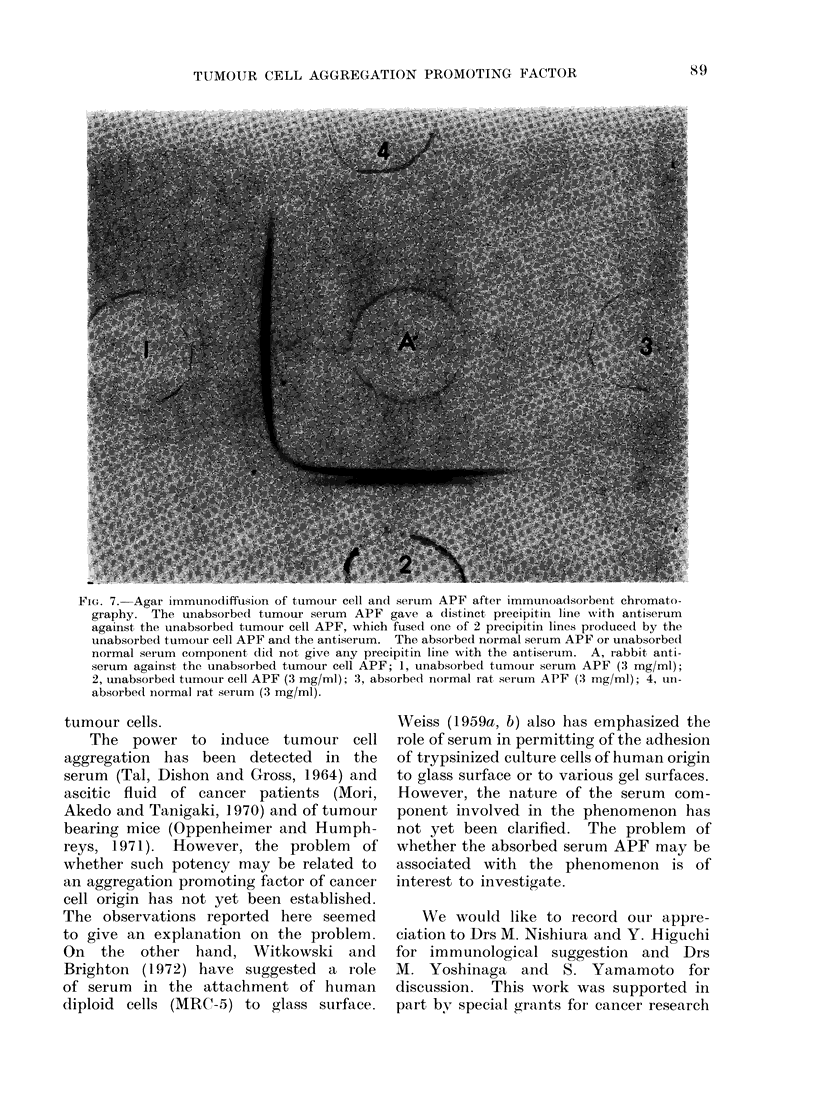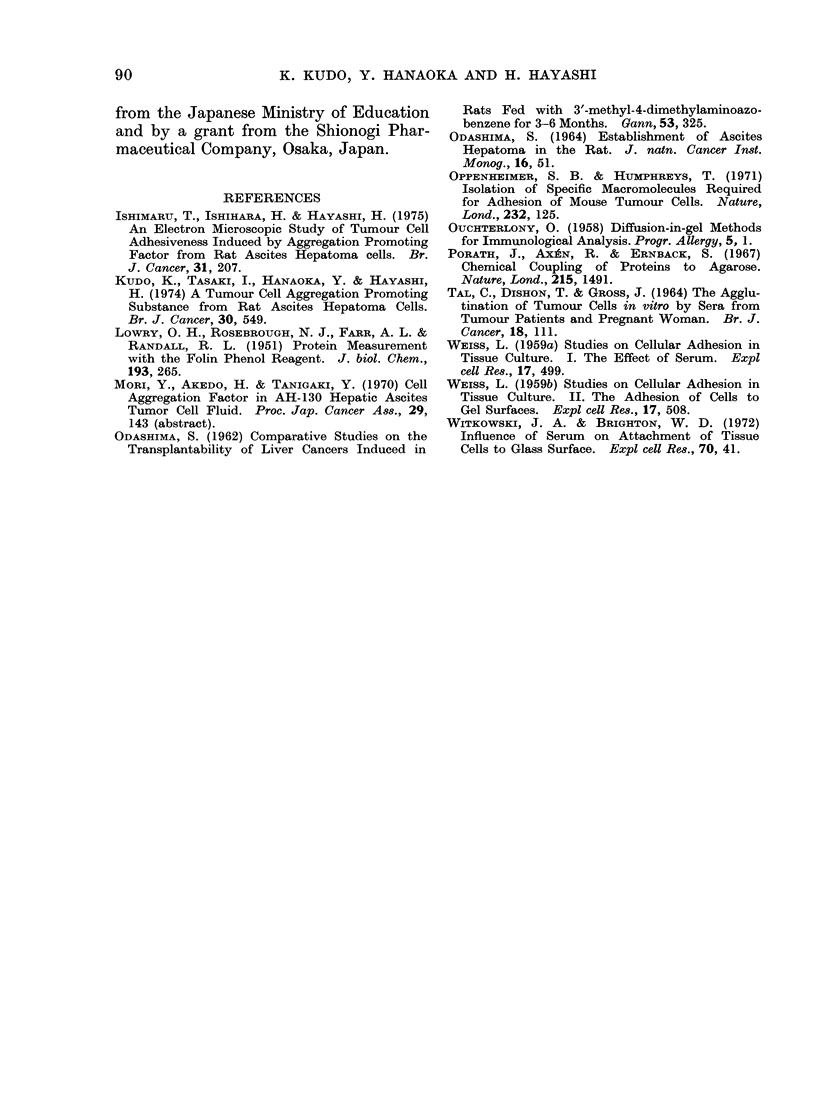# Characterization of tumour cell aggregation promoting factor from rat ascites hepatoma cells: Separation of two factors with different antigenic property.

**DOI:** 10.1038/bjc.1976.8

**Published:** 1976-01

**Authors:** K. Kudo, Y. Hanaoka, H. Hayashi

## Abstract

**Images:**


*Br. J. Cancer* (1976) **33**, 79

### CHARACTERIZATION OF TUMOUR CELL AGGREGATION PROMOTING FACTOR FROM RAT ASCITES HEPATOMA CELLS: SEPARATION OF TWO FACTORS WITH DIFFERENT ANTIGENIC PROPERTY*

K. KUDO, Y. HANAOKA AND H. HAYASHI

*From the Department of Pathology, Kumamoto University Medical School, Kumamoto 860, Japan*

Received 14 May 1975 Accepted 11 September 1975

**Summary.—The previously described glycoprotein that promotes tumour cell aggregation, derived from rat ascites hepatoma cells and capable of partial purification by chromatography, was found to be a mixture of 2 factors with different antigenic property. One was not absorbed by immunoadsorbent chromatography with anti-rat serum antibody and the other was. The action of the unabsorbed factor was clearly more potent than that of the absorbed factor. Both the factors were found in the serum of tumour bearing rats and the action of the unabsorbed factor was also more potent than that of the absorbed factor; its amount increased with time after i.p. inoculation of the cells. The serum of healthy rats contained the absorbed factor but not the unabsorbed factor. It was thus assumed that the unabsorbed factor was associated with the hepatoma cell surface itself and released into the serum, while the absorbed factor was associated with serum protein coating the cell.**

As PREVIOUSLY described (Kudo *et al.*, 1974), a substance capable of inducing tumour cell aggregation, which is probably a glycoportein with no cytotoxicity, has been separated from rat ascites hepatoma cells and partially purified by chromatography. The thermostable substance was clearly effective for rat ascites hepatoma cells as well as SV40 transformed cells, but not for normal rat liver cells and red blood cells. The action of this material was more potent than that of Jack bean concanavalin A (con A) and it was suggested that this material may have a binding site on the cell surface, different from the site for con A, because its effect was not influenced by con A inhibitors. Adhesiveness of rat ascites hepatoma cells induced by this substance was characterized by gradual development of known binding structures; simple apposition and intermediate junction developed in the early stage, and desmosome and focal tight junction in the later stage (Ishimaru, Ishihara and Hayashi, 1975). It was thus assumed that the substance might be involved in the development of such binding structures as a triggering mechanism of tumour cell adhesiveness. The purpose of the present communication is to record the evidence that the substance described above is a mixture of 2 tumour cell aggregation inducing factors with different antigenic property; the one may be associated with hepatoma cell surface and the other with serum protein coating the cell surface.

### MATERIALS AND METHODS

*Rat ascites hepatoma.*—Rat ascites hepatoma AH136B (Odashima, 1962) and AH109A (Odashima, 1964) have been maintained in our laboratory by routine 10-day interval passage of $1 \times 10^6$ AH136B cells or by routine weekly passage of $2 \times 10^6$ AH109A cells injected i.p. into 80–100 g male rats of the Donryu strain. The majority (about 98%) of AH136B cells were found to form cell islands of varying size *in vivo*. On the other hand, most (about 98%) of AH109A cells were found to be free *in vivo*.

* This is No. 3 of the studies on tumour cell aggregation promoting factor.

*In vitro assay for cell aggregation.*—This was performed essentially by the method previously described (Kudo *et al.*, 1974). One ml of the test sample at the same concentration (absorbancy 0·5 at 280 nm/ml) was mixed with 1 ml of cell suspension in a Falcon tube (1·5 × 9·5 cm) and incubated at 37°C in a roller tube culture apparatus, model Te-Her (Hirasawa Co., Tokyo, Japan) run at one rotation/8 min. At 30 min after incubation, cell aggregation in both gross and microscopic features was recorded. The grading of the induced cell aggregation was achieved by counting the aggregating cells and floating cells respectively in the fluid at 30 min of incubation, at which macroscopic cell aggregation became satisfactory. The intensity of cell aggregation was roughly graded as follows: ++, over 70 ± 5% of originally suspended cells were aggregated; +, over 50 ± 5% aggregated; ±, over 30 ± 5% aggregated; and –, below 20% aggregated. All the samples described below were tested after dialysis against Hanks' balanced salt solution for 12 h.

*Preparation of cell suspension.*—AH109A cell suspension was prepared as follows (Ishimaru *et al.*, 1975): the ascites fluid (20 ml) withdrawn by i.p. puncture 7 days after inoculation of AH109A cells and diluted 1 : 5 with 0·45% NaCl solution. After washing with 3 changes of 0·45% NaCl and 2 changes of Hanks' balanced salt solution, tumour cells were sedimented by centrifugation at 25 *g* for 10 min. The cells were finally suspended at a concentration of $1{\cdot}5 \times 10^6$ cells/ml in Hanks' balanced salt solution. As previously described (Ishimaru *et al.*, 1975), the aggregation promoting factor, separated from AH136B cells, showed a similar effect for aggregation of AH109A cells when tested at concentration of $5 \times 10^5$ cells/ml (Kudo *et al.*, 1974). The cell suspension was convenient for the present experiment because the majority of AH109A cells were present in a free form *in vivo*. On the other hand, AH136B cells needed previous dissociation, because of *in vivo* island formation of the cells (Kudo *et al.*, 1974).

*Isolation of aggregation promoting factor from tumour cells (tumour cell APF).*—This was carried out essentially by the method previously described (Kudo *et al.*, 1974). APF was released from $15 \times 10^8$ AH136B cells, suspended in Hanks' balanced salt solution (free of calcium and magnesium), received 50 gentle pipettings and was then allowed to stand for 3 h in the cold (0°C). The supernatant fluid, obtained after centrifugation at 300 *g* for 10 min and then at 10,000 *g* for 30 min, was filtered through Millipore filters (pore size 0·3 μm). The supernatant fluid (10–20 ml, absorbancy 5–7 at 280 nm/ml) was applied to a column (2·0 × 20·0 cm) of DEAE-Sephadex equilibrated with 0·02 mol/l phosphate buffer (pH 6·8). The flow rate was 20 ml/h and 5 g effluent fractions were collected. The second peak (eluted in 0·02 mol/l phosphate buffer plus 0·3 mol/l NaCl on DEAE-Sephadex; 5 ml, absorbancy 4–6 at 280 nm/ml) was placed on a Biogel A-5m column (2·0 × 90·0 cm) equilibrated with Hanks' balanced salt solution. Filtration was performed at a rate of 5 drops/min and 4 g effluent fractions were collected. The second component was used as tumour cell APF.

*Preparation of antisera and antibody.*—Antisera against rat serum were prepared in rabbits (2·5–3·0 kg) by repeated intradermal injections of healthy rat serum (0·5 ml, once per week, 6–8 weeks) with Freund's complete adjuvant (Difco, Detroit, Michigan, U.S.A.). Only antisera with titres of $2^7$–$2^8$, measured by routine precipitin test, were used. From the antisera, the antibody fraction was separated at 33% saturation with ammonium sulphate. Antisera against tumour cell APF or unabsorbed tumour cell APF, as described below, were respectively prepared in rabbits (2·5–3·0 kg) by repeated intradermal injections of each APF (3 mg, once per week, 6–8 weeks) with Freund's complete adjuvant; intradermal injections, in divided dose, were given at 30 sites in the back, abdomen and proximal limbs of the animals. One week later, a booster injection (3 mg APF in 20 ml of physiological saline containing 0·5 mg antihistamine) (Polaramine, Schering Co., Bloomfield, New Jersey, U.S.A.) was given, followed by a similar injection one week later. Only antisera with titres of $2^6$–$2^7$, measured by routine precipitin test, were used.

*Preparation of immunoadsorbent column with Sepharose 4B coupled with anti-rat serum antibody.*—The antibody fraction, separated from rabbit antisera against normal rat serum, was coupled with Sepharose 4B (Pharmacia, Uppsala, Sweden) according to the method of Porath, Axén and Ernback (1967). At room temperature, 40 ml of cyanogen bromide (25 mg/ml in distilled water) were added to a volume of Sepharose 4B suspension corresponding to 1·0 g Sepharose. The pH was rapidly adjusted to 11 and kept constant for 6 min by continuous addition of 2·0 mol/l sodium hydroxide. The reaction mixture was gently stirred during the process. The gel was immediately washed on a glass filter with ice water, and mixed with the antibody fraction (800 mg) which was dialysed against 0·1 mol/l sodium hydrogen carbonate for 16 h at 4°C. After sitrring gently for 16 h at 4°C, the mixture was washed on a glass filter with 0·1 mol/l sodium hydrogen carbonate to remove free protein and then with 0·02 mol/l phosphate buffer (pH 6·8).

*Estimation of protein concentration.*—This was performed by the method of Lowry *et al.* (1951) using bovine serum albumin as a standard. However, in the course of chromatography, protein concentrations were shown by the absorbancy at 280 nm/ml.

### RESULTS

### I. *Separation of two different aggregation promoting factors from tumour cells by immunoadsorbent chromatography*

(a) *Immunodiffusion analysis of tumour cell APF.*—Since it was supposed that ascites hepatoma cells were coated with serum protein and retained the protein in spite of careful washing of the cells, immunological assay with rabbit antisera against APF was performed on the APF. Agar immunodiffusion, pH 8·6, $\mu$m = 0·1, was carried out according to the method of Ouchterlony (1958). The tumour cell

FIG. 1.—Agar immunodiffusion of tumour cell APF eluted on Biogel. The APF gave 2 distinct precipitin lines, one weak precipitin line and a diffusely precipitated faint mass with rabbit antiserum against the APF. Normal serum APF eluted on Biogel gave 3 weak precipitin lines and a diffusely precipitated faint mass with the antiserum noted above. A, rabbit antiserum against tumour cell APF; 1 and 2, tumour cell APF (3 mg/ml); 3 and 4, normal serum APF (10 mg/ml).

APF gave 2 distinct precipitin lines, one weak precipitin line, and diffusely precipitated faint mass when tested with rabbit antiserum against the APF (1, 2 in Fig. 1). On the other hand, the normal serum APF, which was similarly eluted on DEAE-Sephadex and then on Biogel, as described below, gave 3 weak precipitin lines and diffusely precipitated faint mass when tested with the same antiserum as mentioned above (3, 4 in Fig. 1). Obvious fusion between the lines by tumour cell APF and serum APF was not revealed. It was thus suggested that tumour cell APF sample contained some components of serum.

(b) *Immunoadsorbent chromatography of tumour cell APF.*—The APF from tumour cells was concentrated under vacuum pressure dialysis to give an adsorbancy 2–3 at 280 nm/ml. Five ml of the concentrated APF were applied to a column (2·0 × 8·0 cm) of Sepharose 4B coupled with rabbit anti-rat serum antibody previously equilibrated with 0·02 mol/l phosphate buffer (pH 6·8). Elution was done by change of eluting buffers as follows: (1) 0·02 mol/l phosphate buffer (pH 6·8) and (2) 1·0 mol/l acetic acid (pH 2·4). The flow rate was 18 ml/h and 5 *g* effluent fractions were collected. The total yield was about 99% of the applied sample measured as the absorbancy at 280 nm; the first comprised 67% and the second 32%. The first (unabsorbed) component was clearly potent for aggregation of AH109A cells (Fig. 2a). On the other hand, the potency of the second (absorbed) component was apparently less marked (Fig. 2a). In order to avoid a problem due to column overloading, the first (unabsorbed) component was re-chromatographed under the same conditions as mentioned above. The recovery of the applied sample was about 97% and concentrated in the first (unabsorbed) component; the component was similarly active (Fig. 2b). When the assay was done by agar immunodiffusion with rabbit antiserum against the unabsorbed component, *i.e.*, unabsorbed tumour cell APF, the unadsorbed APF gave only 2 distinct precipitin lines (1, 2 in Fig. 3), but the normal serum APF (eluted on Biogel) did not give any precipitin line (3, 4 in Fig. 3). It was thus suggested that the unabsorbed tumour cell APF may have the different antigenic property with normal serum APF.

II. *Separation of 2 different aggregation promoting factors from tumour bearing rat serum by immunoadsorbent chromatography*

(a) *Detection of aggregation promoting factors in the serum of normal and tumour bearing rats.*—The serum was freshly harvested from healthy and tumour bearing rats (at 6 and 9 days after i.p. inoculation of 1 × $10^6$ AH136B cells) respectively. According to the method previously described (Kudo *et al.*, 1974), each serum sample (3–4 ml, absorbancy 40–60 at 280 nm/ml) was applied to a column (2·0 × 20·0 cm) of DEAE-Sephadex A-50 equilibrated with 0·02 mol/l phosphate buffer (pH 6·8), and eluted by stepwise concentration changes of eluting buffers. APF, eluted in 0·02 mol/l phosphate buffer plus 0·3 mol/l NaCl, was further eluted on Biogel equilibrated with Hanks' balanced salt solution; and the second component eluted was used as the serum APF. APF activity of tumour bearing rat serum was more potent than that of healthy rat serum, and seemed to increase gradually according to the duration after i.p. inoculation of the cells (Table).

(b) *Immunoadsorbent chromatography of normal and tumour serum APF.*—The APF samples from the sera of healthy and tumour bearing rats were concentrated under vacuum pressure dialysis to give an absorbancy 3–4 at 280 nm/ml. Five ml of the concentrated samples were applied to an immunoadsorbent column (2·0 × 8·0 cm) prepared by the same method as mentioned above and eluted. The flow rate was 18 ml/h and 5 *g* effluent fractions were collected.

FIG. 3.—Agar immunodiffusion of unabsorbed tumour cell APF after immunoadsorbent chromatography. The APF gave only 2 distinct precipitin lines with rabbit antiserum against the APF, showing disappearance of one weak precipitin line and diffusely precipitated mass demonstrated in Fig. 1. Normal serum APF did not give any precipitin line with the antiserum. A, rabbit antiserum against unabsorbed tumour cell APF after immunoadsorbent chromatography; 1 and 2, unabsorbed tumour cell APF (3 mg/ml); 3 and 4, normal serum APF eluted on Biogel (10 mg/ml).

TABLE.—*Aggregation Promoting Effect of Serum of Healthy and Tumour Bearing Rats*

| Dilution of samples | Factor from normal rat serum | Factor from AH136B bearing rat serum* | Factor from AH136B bearing rat serum† |
|---|---|---|---|
| 1× | + | + | + |
| 2× | + | + | + |
| 4× | – | + | + |
| 8× | – | – | + |
| 16× | – | – | – |
| Medium | – | – | – |

Each sample was obtained after gel filtration on Biogel and tested at the same concentration (absorbancy 0·5 at 280 nm/ml).

* Serum was obtained 6 days after i.p. inoculation of tumour cells.

† Serum was obtained 9 days after i.p. inoculation of tumour cells.

The total yield was about 99% of the applied tumour serum APF samples, measured as the absorbancy at 280 nm; the first comprised 42% and the second 57% (Fig. 4a). The first component was more potent for AH109A cell aggregation than the second component. The first component was re-chromatographed under the same conditions as described above. The recovery of the applied sample was about 99%; the first comprised 57% and the second 42% (Fig. 4b). The first component was more active than the second component. In order to obtain precise results, the first component was further re-chromatographed under the same conditions as noted above. The

FIG. 4c.—Further re-chromatography of unabsorbed tumour serum APF under the same conditions as mentioned above. APF activity was assayed at the same concentration (absorbancy 0·5 at 280 nm/ml).

recovery of the applied sample was about 98% and concentrated in the first component; the component was clearly active (Fig. 4c). On the other hand, APF activity of normal rat serum, although apparently less marked, was found only in the second (absorbed) component (Fig. 5).

When the APF samples after re-chromatography were tested by agar immunodiffusion, the unabsorbed tumour serum APF gave only one distinct precipitin line with antiserum against the unabsorbed tumour cell APF noted above (2 in Fig. 6), but the absorbed tumour serum APF produced no precipitin line with the same antiserum (1 in Fig. 6), indicating that the unabsorbed tumour serum APF shares antigenic sites common with the unabsorbed tumour cell APF, but not with the adsorbed tumour serum APF. On the other hand, the absorbed tumour serum APF gave 2 distinct precipitin lines and one weak precipitin line with antiserum against normal rat serum (3 in Fig. 6), but the unabsorbed tumour serum APF did not give any precipitin line with the antiserum (2 in Fig. 6). Further agar immunodiffusion analysis confirmed the following: the unabsorbed tumour serum APF gave only one distinct precipitin line with antiserum against the unabsorbed tumour cell APF noted above (1 in Fig. 7), which fused obviously one of 2 precipitin lines produced by the unabsorbed tumour cell APF and the antiserum (2 in Fig. 7). The absorbed normal serum APF (3 in Fig. 7) or the unabsorbed normal serum component (4 in Fig. 7) produced no precipitin line with the antiserum described above, indicating that the absorbed normal serum APF has the different antigenic property with the unabsorbed tumour cell and serum APF.

FIG. 5.—Immunoadsorbent chromatography of normal serum APF eluted on Biogel. Each effluent fraction was tested at the same concentration (absorbancy 0·5 at 280 nm/ml) for aggregation activity.

DISCUSSION

The observations presented here demonstrated that a tumour cell APF, isolated from rat ascites hepatoma cells by the method previously described (Kudo *et al.*, 1974), was a mixture of at least 2 factors with different antigenic determinants. The one, which is clearly more potent for induction of AH109A cell aggregation, was not absorbed by immunoadsorbent chromatography with anti-rat serum antibody, *i.e.*, unabsorbed tumour cell APF. On the other hand, the other, which is apparently less active, was absorbed by the antibody, *i.e.*, absorbed tumour cell APF. On the basis of observations that tumour cells used were floating in the ascitic fluid *in vivo*, it was assumed that the unabsorbed tumour cell APF may be related to the surface protein of tumour cells themselves, but the absorbed tumour cell APF to the serum protein coating the tumour cell surface. In this connection, it was important to note that the serum of tumour bearing rats contained 2 tumour cell aggregation promoting factors, which could be differentiated by immunoadsorbent chromatography with anti-rat serum antibody, *i.e.*, absorbed and unabsorbed tumour serum APF. The unabsorbed tumour serum APF was also more potent for induction of AH109A cell aggregation than the absorbed APF,

FIG. 6.—Agar immunodiffusion of tumour serum APF after immunoadsorbent chromatography. The unabsorbed tumour serum APF gave one distinct precipitin line with rabbit antiserum against the unadsorbed tumour cell APF, but did not produce any precipitin line with rabbit antiserum against normal rat serum. The absorbed tumour serum APF produced 2 distinct and one weak precipitin lines with antiserum against normal rat serum, but not with antiserum against the unabsorbed tumour cell APF. A, rabbit antiserum against unabsorbed tumour cell APF; B, rabbit antiserum against normal rat serum; 1 and 3, absorbed tumour serum APF (3 mg/ml); 2, unabsorbed tumour serum APF (3 mg/ml).

as confirmed on the unabsorbed tumour cell APF. Since the unabsorbed tumour serum APF shared the antigenic sites common with the unabsorbed tumour cell APF, it was suggested that both the unabsorbed APF may be similar or identical in nature. On the other hand, since the absorbed tumour serum APF had the common antigenic property with the absorbed normal serum APF, but not with the unabsorbed tumour cell APF or the unabsorbed tumour serum APF, it was assumed that both the absorbed APF may be similar or identical in nature but were different from both the unabsorbed APF. The serum of healthy rats contained the absorbed APF but not the unabsorbed APF. Since both the absorbed serum APF from healthy and tumour bearing rats were similarly less active for induction of AH109A cell aggregation, and the potency of the absorbed tumour serum APF was independent of the duration after i.p. inoculation of tumour cells, it was presumed that the unabsorbed APF in tumour bearing rat serum was released from the tumour cell surface; the APF potency was increased gradually according to the duration after inoculation of

FIG. 7.—Agar immunodiffusion of tumour cell and serum APF after immunoadsorbent chromatography. The unabsorbed tumour serum APF gave a distinct precipitin line with antiserum against the unabsorbed tumour cell APF, which fused one of 2 precipitin lines produced by the unabsorbed tumour cell APF and the antiserum. The absorbed normal serum APF or unabsorbed normal serum component did not give any precipitin line with the antiserum. A, rabbit antiserum against the unabsorbed tumour cell APF; 1, unabsorbed tumour serum APF (3 mg/ml); 2, unabsorbed tumour cell APF (3 mg/ml); 3, absorbed normal rat serum APF (3 mg/ml); 4, unabsorbed normal rat serum (3 mg/ml).

tumour cells.

The power to induce tumour cell aggregation has been detected in the serum (Tal, Dishon and Gross, 1964) and ascitic fluid of cancer patients (Mori, Akedo and Tanigaki, 1970) and of tumour bearing mice (Oppenheimer and Humphreys, 1971). However, the problem of whether such potency may be related to an aggregation promoting factor of cancer cell origin has not yet been established. The observations reported here seemed to give an explanation on the problem. On the other hand, Witkowski and Brighton (1972) have suggested a role of serum in the attachment of human diploid cells (MRC-5) to glass surface. Weiss (1959*a*, *b*) also has emphasized the role of serum in permitting of the adhesion of trypsinized culture cells of human origin to glass surface or to various gel surfaces. However, the nature of the serum component involved in the phenomenon has not yet been clarified. The problem of whether the absorbed serum APF may be associated with the phenomenon is of interest to investigate.

We would like to record our appreciation to Drs M. Nishiura and Y. Higuchi for immunological suggestion and Drs M. Yoshinaga and S. Yamamoto for discussion. This work was supported in part by special grants for cancer research from the Japanese Ministry of Education and by a grant from the Shionogi Pharmaceutical Company, Osaka, Japan.

### REFERENCES

ISHIMARU, T., ISHIHARA, H. & HAYASHI, H. (1975) An Electron Microscopic Study of Tumour Cell Adhesiveness Induced by Aggregation Promoting Factor from Rat Ascites Hepatoma cells. *Br. J. Cancer*, **31**, 207.

KUDO, K., TASAKI, I., HANAOKA, Y. & HAYASHI, H. (1974) A Tumour Cell Aggregation Promoting Substance from Rat Ascites Hepatoma Cells. *Br. J. Cancer*, **30**, 549.

LOWRY, O. H., ROSEBROUGH, N. J., FARR, A. L. & RANDALL, R. L. (1951) Protein Measurement with the Folin Phenol Reagent. *J. biol. Chem.*, **193**, 265.

MORI, Y., AKEDO, H. & TANIGAKI, Y. (1970) Cell Aggregation Factor in AH-130 Hepatic Ascites Tumor Cell Fluid. *Proc. Jap. Cancer Ass.*, **29**, 143 (abstract).

ODASHIMA, S. (1962) Comparative Studies on the Transplantability of Liver Cancers Induced in Rats Fed with 3′-methyl-4-dimethylaminoazobenzene for 3–6 Months. *Gann*, **53**, 325.

ODASHIMA, S. (1964) Establishment of Ascites Hepatoma in the Rat. *J. natn. Cancer Inst. Monog.*, **16**, 51.

OPPENHEIMER, S. B. & HUMPHREYS, T. (1971) Isolation of Specific Macromolecules Required for Adhesion of Mouse Tumour Cells. *Nature, Lond.*, **232**, 125.

OUCHTERLONY, O. (1958) Diffusion-in-gel Methods for Immunological Analysis. *Progr. Allergy*, **5**, 1.

PORATH, J., AXÉN, R. & ERNBACK, S. (1967) Chemical Coupling of Proteins to Agarose. *Nature, Lond.*, **215**, 1491.

TAL, C., DISHON, T. & GROSS, J. (1964) The Agglutination of Tumour Cells *in vitro* by Sera from Tumour Patients and Pregnant Woman. *Br. J. Cancer*, **18**, 111.

WEISS, L. (1959*a*) Studies on Cellular Adhesion in Tissue Culture. I. The Effect of Serum. *Expl cell Res.*, **17**, 499.

WEISS, L. (1959*b*) Studies on Cellular Adhesion in Tissue Culture. II. The Adhesion of Cells to Gel Surfaces. *Expl cell Res.*, **17**, 508.

WITKOWSKI, J. A. & BRIGHTON, W. D. (1972) Influence of Serum on Attachment of Tissue Cells to Glass Surface. *Expl cell Res.*, **70**, 41.